# Chronic Expanding Hematoma With Progressive Osteolysis After Total Hip Arthroplasty: A Case Report

**DOI:** 10.7759/cureus.50684

**Published:** 2023-12-17

**Authors:** Sakiyo Abe, Kenichi Oe, Fumito Kobayashi, Tomohisa Nakamura, Takanori Saito

**Affiliations:** 1 Department of Orthopaedic Surgery, Kansai Medical University, Hirakata, JPN

**Keywords:** adverse reaction to metal debris, pseudotumor, total hip arthroplasty, progressive osteolysis, chronic expanding hematoma

## Abstract

A 31-year-old man without any other medical history developed severe hip pain seven years after right primary total hip arthroplasty (THA). Radiography revealed extensive progressive osteolysis around the cup and stem. Periprosthetic infections and adverse reactions to the metal debris were absent. Right revision THA was performed, and chronic expanding hematoma (CEH) was diagnosed based on a comprehensive assessment. CEH should be diagnosed early because progressive osteolysis may generate an extensive hematoma. Thus, it should be considered when progressive osteolysis of an unknown cause is encountered after THA.

## Introduction

Chronic expanding hematoma (CEH) is a slow-growing hematoma that develops over at least one month after trauma or surgery [1]. CEH can occur anywhere in the body but is most common in deep areas of the trunk, including the thoracic and pelvic cavities, or the extremities, involving especially subcutaneous tissue [2-4]. Although there are some theories regarding the cause of CEH, they remain essentially unresolved. In addition, some authors also reported CEH with pseudotumor formation after total hip arthroplasty (THA) [5-10].

Herein, we report a rare case of CEH without pseudotumor formation occurring seven years after THA. To the best of our knowledge, no reports of CEH without pseudotumor formation occurring after THA exist. The diagnosis of early CEH is difficult during the preoperative examination and even based on pathological findings. However, we must consider CEH when progressive osteolysis of an unknown cause is encountered after THA.

## Case presentation

**Figure 1:**
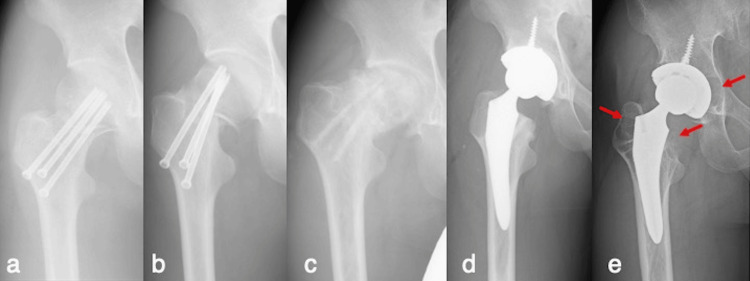
Anteroposterior radiographs of the right hip. (a) After internal fixation for a right femoral neck fracture 10 years ago; (b) femoral head displacement at two weeks; (c) necrotic femoral head after one year. The implant was removed; (d) post right total hip arthroplasty performed after two years; (e) extensive progressive osteolysis after seven years (arrows).

The patient was a 31-year-old man (weight, 71.6 kg; height, 180 cm; body mass index, 22.1 kg/m^2^). Ten years ago, he sustained a right femoral neck fracture while playing tennis and underwent internal fixation (Figure 1a). Two weeks later, the femoral head was displaced; however, no additional surgery was performed (Figure 1b). The femoral head became necrotic, and the screws were removed one year postoperatively (Figure 1c). Two years postoperatively, right THA (Cup: SQRUM HA; Head: 36-mm ceramic head; Stem: J-taper, KYOCERA Medical, Osaka, Japan) was performed for osteonecrosis of the femoral head (Figure 1d). The patient continued playing tennis as a hobby after the THA. Seven years later, he experienced severe hip pain and was diagnosed with myalgia at the same hospital. Since the pain did not improve with the treatment provided, the patient consulted us. He had no other medical or medication history. The patient had difficulty walking due to right hip pain. The wound was intact. Radiography revealed extensive progressive osteolysis around the cup and stem (Figure 1e). 

**Figure 2:**
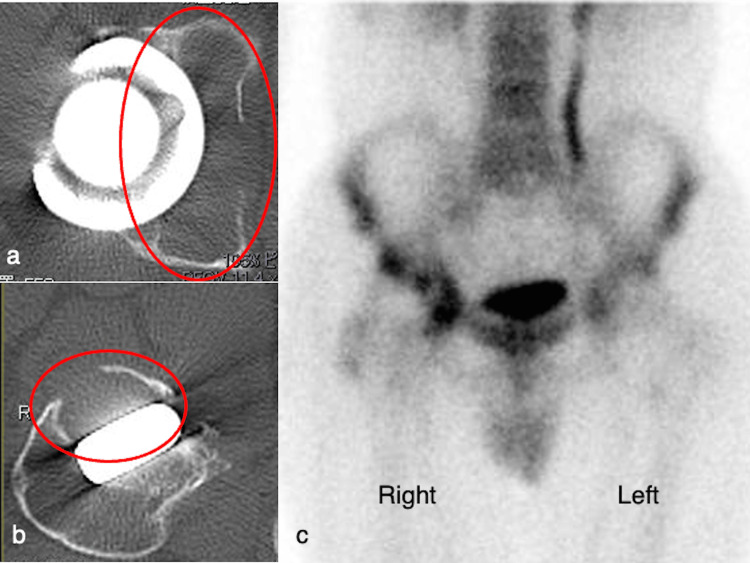
Plain computed tomography and bone scintigraphy findings. (a, b) The computed tomography images show severe osteolysis around the cup and stem; (c) the bone scintigraphy image shows uptake on the acetabular side.

Plain computed tomography (CT) showed similar osteolysis without mass effect formation, and bone scintigraphy revealed uptake on the acetabular side (Figure 2). There was no hematoma formation around the acetabulum or iliopsoas. Preoperative laboratory examination showed a white blood cell count of 5,200/mm^3^, a hemoglobin level of 14.4 g/dL, a platelet count of 19.9 × 103/μL, and a serum C-reactive protein level of 0.13 mg/dL. Serum metal ion concentrations were not assessed. The aspirated joint fluid was bloody; however, no pathogens were detected. Preoperative angiography and embolization were not performed because the lesion did not invade the pelvic cavity. No culture-positive periprosthetic joint infection (PJI) or adverse reaction to the metal debris (ARMD) was suspected, and the diagnosis remained unknown during preoperative evaluations.

**Figure 3:**
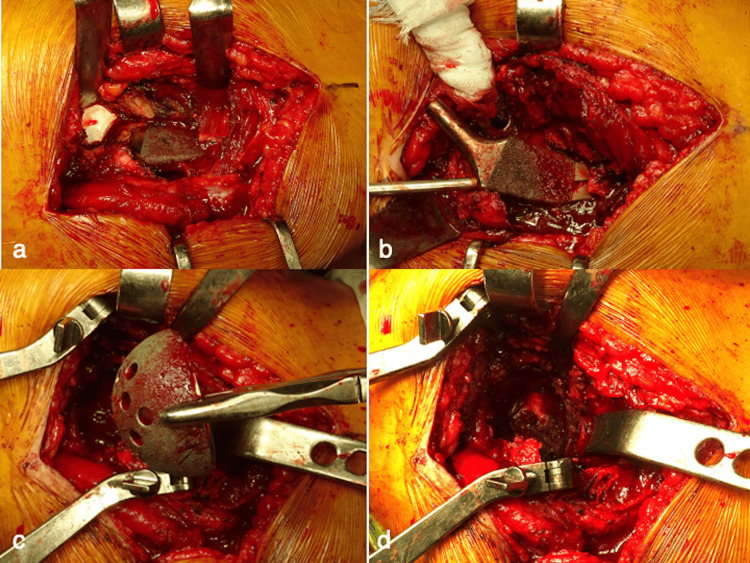
Intraoperative photographs. (a) Extensive osteolysis at the proximal femur; (b) stem removal; (c) cup removal; (d) thin acetabular floor showing hematoma with capsular formation.

**Figure 4:**
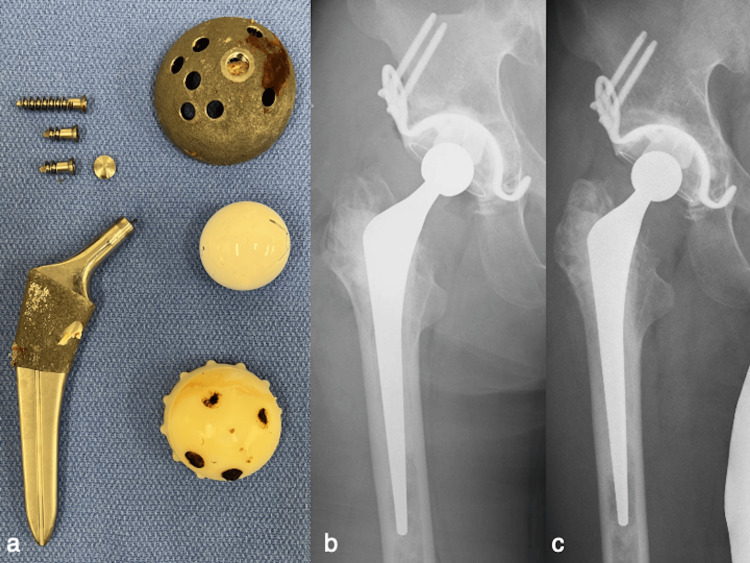
Postoperative findings. (a) Implants show no wear or deformity; (b) the radiograph immediately after the revision; (c) the radiograph obtained one year after the revision shows no recurrent osteolysis.

**Figure 5:**
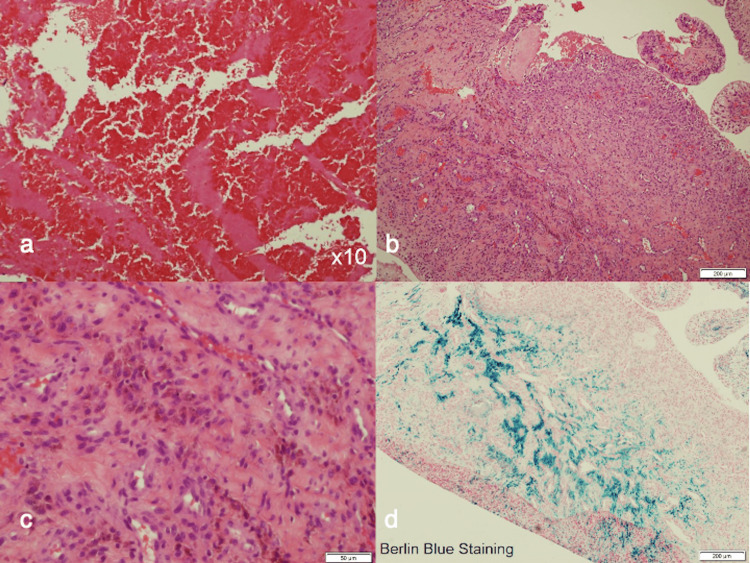
Pathological findings. (a) Hematoxylin and eosin-stained section shows blood clots (×10); (b) no lymphocytic infiltration or metallosis is present; (c) some foreign bodies likely comprising polyethylene are observed; (d) berlin blue-stained section shows hemosiderin deposition.

The patient underwent revision THA. A transgluteal approach in the lateral position was used [11], and a massive hematoma was found during the osteotomy of the greater trochanter. Stem removal was easy because severe osteolysis and loosening had occurred in the proximal femur (Figures 3a, 3b). Cup removal was also easy (Figure 3c). The acetabular floor was thin, and a hematoma with capsular formation was observed (Figure 3d). The periprosthetic tissues showed no suspicious findings of infection or metal debris, and the removed implants revealed no wear and damage (Figure 4a). A one-stage revision THA (KT plate; K-MAX CLHO flanged cup; 26-mm metal head; SC long stem; PALACOS G bone cement, KYOCERA Medical) was performed using the endofemoral shooting technique [12]. In the absence of cortical bone, the strut and mushed allografts were augmented using ETHIBOND (Johnson & Johnson K.K., Tokyo, Japan) [12]. Intraoperative cultures of joint fluid, tissues, and sonicated implants did not reveal any bacteria [13]. The partially resected trochanteric fragment was reattached to the greater trochanter using two ultra-high molecular polyethylene fiber cables (NESPLON Cable System, Alfresa Pharma Co., Osaka, Japan) [11]. Pathological examination revealed blood clots and foreign bodies, likely comprising polyethylene. No lymphocytic infiltration or metallosis was observed. Berlin blue staining revealed hemosiderin deposition (Figure 5). PJI and ARMD were negative. Ultimately, CEH was diagnosed based on a comprehensive assessment. On the second postoperative day, full weight bearing was allowed. The patient was not allowed to participate in any sports for three months. One year after the revision of THA, the patient had no symptoms, and radiography showed no recurrent osteolysis (Figure 4b). The patient was informed that his data and images would be submitted for publication, and he provided consent for publication.

## Discussion

CEH was first reported by Friedlander et al. in 1968, and Reid et al. defined it as “CEH is a slowly enlarging lesion that persists for more than one month after the initiating events” [1,14]. There are two proposed mechanisms for the development and expansion of such lesions: (1) blood breakdown products create an osmotic gradient that draws more fluid into the hematoma [15], and (2) chronic expansion results from inflammation that is stimulated by blood and its breakdown products (a theory proposed to explain the pathogenesis of subdural hematomas) [16]. Goddard et al. also reported that this inflammatory process might damage the capillaries located in the wall of the mass, leading to recurrent bleeding and CEH [5]. Although hematoma formation can be caused by surgery, trauma, and even minor external forces, the fundamental cause of CEH remains unclear [3,4,14,16].

When diagnosing CEH after THA, it is important to distinguish it from PJI, ARMD, and malignant tumors. Dynamic CT shows a “rim enhancement” during the arterial phase, and T2-weighted magnetic resonance imaging (MRI) shows a “mosaic sign” [17,18]. Specific findings of CEH include overall heterogeneous signal intensity in the soft tissue mass, unlike the homogenous cystic mass that has been reported as ARMD [19]. However, some authors have reported that distinguishing CEH from ARMD or malignant tumors using imaging findings is difficult because a variety of findings based on the phases of CEH exist [6,8,20]. Pseudotumor formation after THA is commonly caused by ARMD, and the pseudotumor occurring after metal-on-metal THA should be carefully diagnosed using MRI and assessment of serum metal ion concentrations [6]. Furthermore, three histological features of CEH have been identified: (1) a peripheral wall of dense fibrous tissues containing (2) fresh and altered blood and (3) a middle zone of loose connective tissues [14]. Additionally, previous studies have demonstrated the deposition of hemosiderin and foreign bodies in the hematoma [5,7]. In the present case, detailed imaging was not performed because the patient had extensive osteolysis without pseudotumor formation on plain CT, and CEH was not suspected. However, in retrospect, assessment using dynamic CT and MRI should have been performed to avoid the potential consequences of delayed diagnosis. The histological findings demonstrated hematoma, foreign bodies, and hemosiderin deposition without lymphocytic infiltration or metallosis, although the characteristic peripheral walls of dense fibrous tissue and the middle zone of loose connective tissue were not observed. Even the pathological evaluations were not definitive, and the diagnosis of early CEH after THA could only be made comprehensively.

The published reports on CEH after THA are presented in Table 1. The duration to onset ranged widely (4-23 years), a variety of implant materials were involved, and THA types included primary and revision THA. There were no obvious histories of trauma. Matsuda et al. hypothesized that osteolysis with hematoma was associated with an inflammatory reaction to hydroxyapatite debris, which was used in impaction bone grafting [7]. However, the potential causes and risk factors for CEH after THA could not be compared because there are few relevant case reports. In the present case, the duration to onset was seven years after primary THA, but it was difficult to accurately point out when exactly CEH occurred. Postoperative activities of the patient might have been associated with CEH formation, whereas other studies indicated no specific causes of CEH. In most patients with high activity levels after THA, the hematoma is usually absorbed. The mechanism by which hematoma is not absorbed and results in CEH remains unknown. The resection of the pseudotumor has been mostly performed for the treatment of CEH after THA; nevertheless, we believe that it is crucial to perform the revision of both cup and stem for extensive osteolysis as much as possible - despite no obvious loosening of implants -because the mechanisms and causes of CEH after THA remain unknown. Furthermore, to the best of our knowledge, CEH without pseudotumor formation after THA has not been previously reported. In the early stage of CEH, the extension of hematoma to soft tissue does not occur; therefore, it is especially difficult to diagnose it preoperatively. Revision THA should be performed in cases where an extensive hematoma has not yet formed.

**Table 1:** Published reports of chronic expanding hematoma after primary or revision THA. F: female, M: male, THA: total hip arthroplasty.

Authors	Year	Age/sex	Duration to reoperation	Implant	Reoperation
Goddard et al. [5]	2011	59/F	Nine years after revision THA	Cementless (S-ROM cup, unknown stem)	Resection arthroplasty
Ando et al. [6]	2017	76/F	Four years after primary THA	Cementless (M2a-Magnum Large Metal Articulation)	Revision (only cup)
Matsuda et al. [7]	2018	79/F	Four years after revision THA	Cementless (Secure-Fit PSL cup, Omnifit stem)	Revision (cup and stem)
Yamashita et al. [8]	2021	84/F	Twenty-two years after primary THA	Cementless (Not described)	Revision (cup and megaprosthesis)
Ishikawa et al. [9]	2021	60/F	Twenty-three years after primary THA	Cement (CP socket, unknown stem)	Revision (only cup)
Ishikawa et al. [9]	2021	47/F	Twenty-three years after primary THA	Cement (Not described)	Revision (cup and stem)
Yamamuro et al. [10]	2022	67/M	Twenty-two years after primary THA	Cementless (Not described)	Revision (cup and megaprosthesis)
Current report	2023	31/M	Seven years after primary THA	Cementless (SQRUM HA cup, J-taper stem)	Revision (cup and stem)

## Conclusions

In the present case, hemorrhage triggered by minor trauma likely caused hematoma formation and bone destruction (early stage of CEH). Although the hematoma was not as large as those reported by previous researchers, it might have expanded further in the future. Diagnosis of early CEH is difficult both preoperatively and intraoperatively, but CEH must be considered when progressive osteolysis of an unknown cause is encountered after THA. Further studies are required because the potential causes and risk factors for CEH after THA are still unknown.
